# Perfluorooctanesulfonic acid contributes to primary open-angle glaucoma in a FABP4-Dependent manner: a novel mechanism for environmental risk of glaucoma

**DOI:** 10.3389/fgene.2026.1807152

**Published:** 2026-05-12

**Authors:** Jiangpeng Lin, Yichi Zhang, Yunzepeng Li, Nan He, Yang Liu, Hui Zheng

**Affiliations:** Department of Ophthalmology, The Fifth Affiliated Hospital, Sun Yat-sen University, Zhuhai, China

**Keywords:** PFOS, POAG, network toxicology, FABP4, HTMC senescence

## Abstract

**Background:**

Primary open-angle glaucoma (POAG) is the most prevalent form of glaucoma globally, with environmental factors increasingly recognized as critical determinants of its onset and progression. However, the potential association between perfluorooctanesulfonic acid (PFOS) exposure and POAG remains poorly understood, and its underlying molecular mechanisms have yet to be elucidated.

**Methods and results:**

Utilizing cross-sectional data from the NHANES database, we identified a significant positive correlation between serum PFOS levels and glaucoma prevalence. The robustness of our study population selection and the focus on POAG were further validated through the Global Burden of Disease (GBD) database and systematic literature review. Network toxicology analysis identified 20 PFOS-exposure-related genes in POAG, with functional enrichment highlighting the biosynthesis of unsaturated fatty acids as a key pathway. Integrated machine learning and bioinformatic analysis pinpointed FABP4 as a pivotal candidate gene. Molecular docking and dynamics simulations confirmed stable binding affinity between PFOS and the FABP4 protein. *In vitro* experiments using human trabecular meshwork cells (HTMCs) demonstrated that PFOS exposure induced cellular senescence, as evidenced by SA-β-gal staining. Western blot analysis revealed that PFOS significantly upregulated the expression of FABP4 and the senescence marker P21. Crucially, targeted functional inhibition of the FABP4 protein successfully rescued PFOS-induced senescence and downregulated P21 expression.

**Conclusion:**

These findings provide novel insights into the toxicological profile of PFOS, suggesting that it contributes to POAG pathogenesis by modulating FABP4-mediated cellular senescence. This study offers a theoretical basis for environmental risk assessment and the development of preventive strategies for POAG.

## 1 Introduction

Glaucoma is a leading cause of irreversible blindness worldwide, characterized by the progressive degeneration of retinal ganglion cells. Due to its typically asymptomatic early stages, patients often remain undiagnosed until significant visual field loss occurs (Jayaram et al., 2023). Despite its severity, timely intervention and active treatment can preserve functional vision in the majority of patients (Benjafield et al., 2019). Consequently, identifying modifiable risk factors for early detection and prevention is a public health priority. Epidemiological data estimate that approximately 95 million people are affected by glaucoma globally, with primary open-angle glaucoma (POAG) being the predominant subtype (global prevalence: 3.05%) compared to primary angle-closure glaucoma (0.50%) (Weinreb et al., 2014). The etiology of POAG is multifactorial, involving a complex interplay of genetic predisposition, metabolic dysregulation, and environmental influences (Ma et al., 2024). Recent evidence has underscored the role of environmental pollutants in exacerbating POAG risk. For instance, long-term exposure to ambient air pollution has been linked to increased POAG incidence, particularly in genetically vulnerable individuals, while bone-deposited lead has been identified as a significant risk factor in the U.S. population (Wang et al., 2018; Zahm et al., 2024).

Despite these advancements, the impact of per- and polyfluoroalkyl substances (PFAS), specifically perfluorooctanesulfonic acid (PFOS), on POAG remains an unexplored frontier. PFOS is a persistent organic pollutant formerly ubiquitous in industrial applications, including firefighting foams and stain-resistant coatings. Characterized by extreme environmental stability and a human half-life exceeding 5 years, PFOS readily bioaccumulates through contaminated drinking water and food chains (Steves et al., 2018). Extensive toxicological studies have established that PFOS induces systemic multi-organ damage, including hepatotoxicity, endocrine disruption, and neurotoxicity by acting as a potent metabolic disruptor. This toxicity is primarily mediated through the pervasive generation of reactive oxygen species (ROS), the disruption of lipid homeostasis via PPAR signaling, and the subsequent activation of apoptotic pathways (Liang L. et al., 2022; Wang et al., 2023). Furthermore, recent high-quality toxicological investigations have broadly underscored the profound capabilities of environmental endocrine-disrupting chemicals (EDCs) and associated toxicants to perturb circulating metabolites, trigger severe oxidative stress, and drive disease pathogenesis across diverse systemic conditions (Lin et al., 2025; Lin et al., 2024). Strikingly, this systemic toxicity profile shares a profound pathogenic convergence with the localized etiology of primary open-angle glaucoma (POAG) (Evangelho et al., 2019). In POAG pathophysiology, the progressive senescence of human trabecular meshwork cells (HTMCs) is fundamentally driven by chronic redox imbalance and the accumulation of lipotoxic metabolites, which collectively increase intraocular pressure. Despite these shared pathological hallmarks, specifically oxidative stress and lipid dysregulation, a critical knowledge gap remains regarding the exact molecular conduit that links systemic PFOS exposure to localized ocular senescence. Given that PFOS structurally mimics endogenous fatty acids, we hypothesized that intracellular lipid chaperones might serve as the primary pathological mediators (Birchfield et al., 2025). Consequently, our mechanistic focus naturally converged on Fatty Acid Binding Protein 4 (FABP4), a central regulator of lipid trafficking and inflammatory cascades. We postulate that FABP4 acts as the crucial molecular bridge, directly interacting with PFOS to orchestrate the downstream oxidative and senescent signaling networks that drive POAG progression (Yu et al., 2026).

**FIGURE 1:**
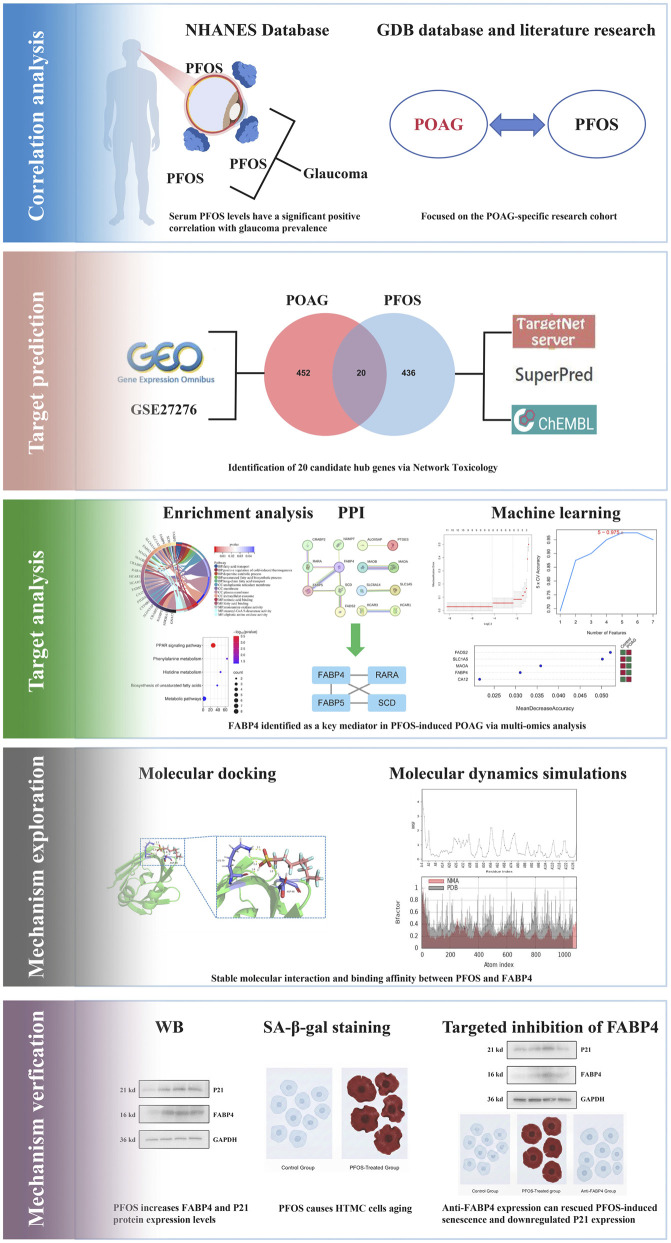
Workflow outlines of the research.

In this study, we employed an integrative approach—combining NHANES epidemiological analysis, GBD database validation, network toxicology, and machine learning—to systematically investigate the association between PFOS and POAG. Our workflow (Figure 1) initially established a statistically significant correlation between serum PFOS levels and glaucoma in the NHANES cohort. We subsequently identified FABP4 as a key mediator through bioinformatic screening and confirmed its binding stability with PFOS via molecular dynamics simulations. Finally, we validated the biological significance of the PFOS-FABP4 axis using a human trabecular meshwork cell (HTMC) senescence model. These findings provide a theoretical foundation for understanding the toxicological mechanisms of PFOS in ocular health and offer scientific support for environmental risk mitigation and POAG prevention.

## 2 Methods

### 2.1 Sample and population

Our research was utilized the data from the surveys conducted during the period from 2005 to 2008, as only these two surveys in all cycles included questions and information on the presence or absence of glaucoma. In this study, 20,497 participants were initially screened out from two NHANES cycles from 2005 to 2008 (Li et al., 2024). The exclusion criteria for the participants are as follows: (Jayaram et al., 2023): A total of 13,317 people under the age of 40 were excluded; (Benjafield et al., 2019); A total of 302 individuals with missing glaucoma disease data were excluded, and the following data were left: Variable VIQ090 (Ever told had glaucoma): including “Yes” and “No”. Variable OPXDGLAU (Glaucoma, right eye) and OPXSGLAU (Glaucoma, left eye): including “No”, “Possible”, “Probable”, “Definite” and “Ungradable”. (Weinreb et al., 2014). Subsequently, 5,914 individuals were excluded from the final analytical cohort due to missing data for essential study variables. Specifically, these exclusions comprised participants lacking valid measurements for serum PFOS concentrations, age, sex, race, marriage, total alcohol consumption, education level, smoking status, body mass index (BMI), and poverty-income ratio (PIR). Ultimately, 1,165 eligible participants were included in the final analysis. A detailed flowchart of the screening process is provided in Supplementary Figure S1.

### 2.2 Covariates assessment

Based on previous studies, we included variables that might affect the outcomes related to glaucoma in our research. Variables such as Age, Sex, Race, Education, PIR, and Marriage were regarded as social demographic factors. Additionally, Body Mass Index (BMI) was listed as an indicator of medical assessment and personal health background. Furthermore, we also considered lifestyle factors, such as smoking status (whether an individual has smoked at least 100 cigarettes in their lifetime) and Total alcohol.

### 2.3 Multivariable analysis

Descriptive statistics were computed for continuous variables as means ± standard error (SE) and for categorical variables as proportions. The distributions of potential confounding variables between glaucoma patients and non-glaucoma people were compared using independent samples t-tests for continuous variables and chi-square tests for categorical variables. To evaluate the association between PFOS and glaucoma, weighted multiple linear regression analyses were performed. Three models were constructed: Model 1 (Crude model): Examined the unadjusted association between PFOS and glaucoma. Model 2: Adjusted for age and sex. Model 3 (Final model): Further adjusted for age, sex, race, marriage, total alcohol consumption (Total.alco), education level, smoking status, body mass index (BMI), and poverty-income ratio (PIR). Additionally, PFOS was categorized into quartiles to assess potential nonlinear trends, and a p value for trend was calculated. All analyses were conducted in R software (version 4.2.3), incorporating appropriate sampling weights to accommodate the complex survey design. All p values with p < 0.05 were believed to be statistically significant. To ensure the national representativeness of the estimates and rigorously account for the complex, multistage, and stratified probability sampling design inherent to NHANES, appropriate survey weights were applied across all statistical analyses. Given the aggregation of two consecutive 2-year survey cycles (2005–2006 and 2007–2008), a new 4-year sample weight variable was constructed by dividing the original 2-year weights WTSC2YR by two, in strict accordance with the official NHANES analytical guidelines. Furthermore, variance estimation was conducted utilizing the Taylor series linearization method. This was operationalized by incorporating the masked variance pseudo-primary sampling unit (PSU) variable (SDMVPSU) and the pseudo-stratum variable (SDMVSTRA) into the survey design models. All weighted analyses were executed using the ‘survey’ package in R software (version 4.2.3).

### 2.4 Utilizing glaucoma epidemiology to assess data selection rigor

The Global Burden of Disease (GBD) study, conducted by the Global Burden of Disease Collaborative Network, provides comprehensive assessments of 371 diseases and injuries (including 40 injuries, 234 non-communicable diseases, and 95 communicable, maternal, neonatal, and nutritional [CMNN] diseases) across 204 countries and territories (Murray and Collaborators, 2024). While numerous studies have used GBD data to estimate glaucoma disease burden, most published literature only includes data up to 2019. For this study, we extracted 2021 glaucoma data from the GBD 2021 study. To validate our age criterion (>40 years) for selecting glaucoma patients in the NHANES database, we obtained glaucoma prevalence and disability-adjusted life years (DALYs) data from the GBD database. Glaucoma is clinically classified into primary open-angle glaucoma (POAG) and primary angle-closure glaucoma (PACG). Due to limitations in accessing complete data from the GEO database, we conducted a literature review which revealed that POAG prevalence substantially exceeds that of PACG. Consequently, we selected POAG as the representative condition for subsequent analyses.

### 2.5 Construction of networks

The chemical structure and Simplified Molecular Input Line Entry System (SMILES) notation of PFOS were obtained from PubChem database (CID: 74483; https://pubchem.ncbi.nlm.nih.gov/). Potential human protein targets of PFOS were identified using three independent databases: ChEMBL (https://www.ebi.ac.uk/chembl/), TargetNet (http://targetnet.scbdd.com/), and SuperPred (https://prediction.charite.de/), with the species parameter restricted to *Homo sapiens*. Following database queries, identified targets were merged and duplicates were removed to generate a non-redundant list of PFOS-associated targets for subsequent analyses. The glaucoma-related gene expression dataset GSE27276 was retrieved from the Gene Expression Omnibus (GEO; https://www.ncbi.nlm.nih.gov/geo/) using the GEOquery package (version 4.2.3) in R software. The dataset comprised 17 glaucoma cases and 19 healthy controls (Zavarzadeh and Abedi, 2023). Differential gene expression analysis was performed using the limma package, with genes meeting the criteria of p < 0.05 and absolute |log2FC|) > 0.58 considered statistically significant. Through systematic integration of the PFOS-associated targets and glaucoma-related differentially expressed genes (DEGs), we identified a subset of overlapping genes that were selected as key candidates for subsequent functional analyses.

### 2.6 Functional enrichment analysis

To elucidate the potential mechanisms by which PFOS contributes to primary open-angle glaucoma (POAG), we performed functional enrichment analysis using Gene Ontology (GO) and the Kyoto Encyclopedia of Genes and Genomes (KEGG) pathway database. GO classification categorizes gene function into three domains:Biological Process (BP), Molecular Function (MF) and Cellular Component (CC). The Database for Annotation, Visualization, and Integrated Discovery (DAVID; v6.8) (https://david.ncifcrf.gov/) was employed to conduct GO and KEGG pathway enrichment analyses (Dennis et al., 2003). P value <0.05 were considered statistically significant.

### 2.7 Protein–protein interaction network analysis

To elucidate the potential key targets of PFOS in the pathogenesis of primary open-angle glaucoma (POAG) and their molecular interactions, the 20 intersecting genes identified in previous analyses were subjected to protein-protein interaction (PPI) network analysis using the Search Tool for the Retrieval of Interacting Genes (STRING) Database (http://string-db.org/) (Szklarczyk et al., 2023). Following the exclusion of genes exhibiting low interaction degrees, a comprehensive PPI network was constructed. For further refinement of core genes, the PPI network data were imported into Cytoscape software for topological analysis. The CytoHubba plugin was employed with the Maximal Clique Centrality (MCC) algorithm to identify the most highly interconnected hub genes.

### 2.8 Identification of core genes through machine learning

Subsequently, an integrative machine learning approach was implemented to enhance the robustness of core gene selection. Least Absolute Shrinkage and Selection Operator (LASSO), an L1-regularized linear regression method, was applied for feature selection. Utilizing the “glmnet” package in R (version 4.2.3), gene expression data from the GSE27276 dataset were analyzed. The optimal model was determined through 10-fold cross-validation to minimize overfitting while maintaining predictive accuracy (Liang Y. et al., 2022). The Support Vector Machine-Recursive Feature Elimination (SVM-RFE) algorithm, a backward selection approach based on the maximum margin principle of support vector machines, was employed to rank genes by their relative importance. This iterative process sequentially eliminates the least contributory features, thereby reducing dimensionality and enhancing model performance. The procedure was repeated until the most discriminative subset of differentially expressed genes (DEGs) was identified (Ning et al., 2023). Random Forest (RF) Analysis, an ensemble learning method renowned for its robustness in high-dimensional data analysis, was implemented with 1000 decision trees to ensure model stability. Feature importance was quantified using the MeanDecreaseAccuracy index, and the optimal number of trees was selected to minimize the out-of-bag error rate. RF was completed using the Wekemo Bioincloud (https://www.bioincloud.tech) (Ghadiri et al., 2022). The candidate genes derived from PPI network analysis, LASSO regression, SVM-RFE, and Random Forest were subjected to intersection analysis. The consensus genes identified through this multi-modal approach represent the most critical molecular targets implicated in PFOS-associated POAG pathogenesis.

### 2.9 Molecular docking of PFOS-FABP4

To investigate the molecular interactions between PFOS and the core gene associated with POAG, we conducted a computational docking analysis. The three-dimensional (3D) crystal structure of the target protein FABP4 (PDB ID: 6LJW) was retrieved from the Protein Data Bank (PDB) (http://www.rcsb.org/), while the structural data for the ligand PFOS (Compound CID: 74483) was obtained from the PubChem Compound Database (https://pubchem.ncbi.nlm.nih.gov/). We used the PyMol software (version 3.1) to preprocess the protein before docking, including hydrogenation (to correct for missing polar hydrogens in crystallographic structures) and removal of water molecules (to eliminate potential interference in binding site prediction). Molecular docking simulations were performed using AutoDock Vina (version 1.1.2), a widely validated tool for predicting ligand-receptor interactions. To encompass the active binding pocket of FABP4, the docking grid box was defined with dimensions of 20 × 20 × 20 Å. The grid center was precisely positioned at the geometric center of the native binding pocket within the 6LJW structure, ensuring comprehensive and unbiased coverage of the internal hydrophobic cavity. Exhaustive conformational sampling to identify optimal binding poses. Selection of the lowest binding energy conformation as the most thermodynamically stable interaction mode. The binding energy (ΔG, kcal/mol) was used as a key metric to evaluate the strength of PFOS-protein interactions. Lower (more negative) binding energies indicate stronger and more favorable binding. The resulting docking conformations were visualized and analyzed in PyMOL software to assess that hydrogen bonding and hydrophobic interactions between PFOS and key amino acid residues. This computational approach provides mechanistic insights into how PFOS may modulate the function of POAG-associated genes (Wang et al., 2021).

### 2.10 Molecular dynamics simulations of PFOS-FABP4

To comprehensively evaluate of the binding affinity between environmental pollutant and protein target, we conducted molecular dynamics (MD) simulations. After preprocessing the protein-ligand complexes to optimize structural configurations using the Pymol software, they were submitted to the CABS-flex server (https://biocomp.chem.uw.edu.pl/CABSflex2/index) and IMODS server (https://imods.iqfr.csic.es/) for molecular dynamics simulations. The CABS-flex 2.0 server simulated trajectories for 10 nanoseconds, yielding 10 structure models and root mean square fluctuation (RMSF) curves for complexes (Wasim et al., 2024). The RMSF analysis quantified residue-specific flexibility, providing insights into local conformational dynamics. To further evaluate the flexibility and rigidity of the structures, we employed the iMODS server for normal mode analysis (NMA). This approach characterized intrinsic protein motions by computing: Deformability: Identifying regions susceptible to structural distortion; B-factors: Comparing theoretical (NMA-derived) and experimental (PDB-based) atomic displacement parameters; Eigenvalues: Quantifying mode stiffness, where lower values indicate higher conformational flexibility; Variances: Evaluating the contribution of individual normal modes to collective dynamics. Together, these analyses elucidated the interplay between pollutant binding and protein structural adaptability, offering a mechanistic perspective on molecular recognition (López-Blanco et al., 2014). To establish a rigorous baseline for structural dynamics, the unliganded (apo) FABP4 protein was subjected to identical MD simulation protocols alongside the PFOS-FABP4 complex. Comparative analyses of RMSF, deformability, and eigenvalues were conducted to quantify the specific ligand-induced conformational stabilizations.

### 2.11 Cell culture and treatment

HTMCs were grown in DMEM/F12 media supplemented with 0.5% penicillin, 0.5% streptomycin, and 15% fetal bovine serum (FBS). They were kept at 37 °C with 5% CO_2_ in a humidified incubator. To keep the cells viable, the culture media was changed every 2 days. When the cells achieved around 90% confluence, they were passaged. To guarantee consistent growth phase entry, the cells were synchronized by serum fasting for a full day prior to any experimental intervention. The experimental groups were as follows: (Jayaram et al., 2023): control group, (Benjafield et al., 2019), H_2_O_2_ group (positive control group), (Weinreb et al., 2014), low-concentration PFOS group (5 μM), and (Ma et al., 2024) high-concentration PFOS group (20 μM). After adding PFOS or culture medium to each group, they were cultured for 72 h and then subsequent experiments were conducted.

### 2.12 Cell viability analysis

We used the Cell Counting Kit-8 (CCK-8) method to detect the survival rate of cells after inoculation in a 96-well plate. 5×10^3^ cells were inoculated in each well. When the HTMC reached 70%–80% fusion, the cells were starved with serum-free DMEM/F12 medium for 24 h. HTM cells were treated with different concentrations of PFOS or H_2_O_2_ (0, 5, 10, 20, 50, 100 and 200 μM) for 72 h, and then co-incubated with CCK-8 for 2 h. The cell viability was determined by measuring the absorbance at 450 nm.

### 2.13 Detection of senescent cells

The primary pathological feature of POAG is the senescence and functional impairment of HTMC. Assessing HTMC senescence serves as a critical indicator to determine whether PFOS induces POAG. To evaluate cellular senescence, HTMCs were first exposed to specified concentrations of PFOS for a duration of 72 h. It is crucial to note that this 72-h period refers strictly to the toxicological exposure phase. Following treatment, senescence-associated β-galactosidase (SA-β-gal) activity was assessed utilizing a fluorescence-based SA-β-gal staining kit (Beyotime Biotechnology, Shanghai, China]), in strict accordance with the manufacturer’s protocol. For the experiment, cells were cultured in 96-well plates, with 5 × 10^3^ cells seeded per well (three technical replicates per group). The detailed procedure was as follows: 1 mL of Senescence-Tracker™ NIR staining working solution was added to each well after being treated with PFOS for 72 h. Cells were incubated at 37 °C for 24 h in a cell culture incubator. Subsequently, the supernatant was aspirated, and cells were washed three times with phosphate-buffered saline (PBS) prior to observation under a fluorescence microscope. The percentage of SA-β-gal-positive cells was quantified using ImageJ software.

### 2.14 Targeted functional inhibition of FABP4 with BMS-309403

To conduct a more in-depth investigation into whether PFOS exerts its cellular effects through FABP4, we utilized the specific competitive inhibitor BMS-309403. Unlike transcriptional suppressors, BMS-309403 specifically targets and blocks the intracellular lipid-binding function and biological activity of the FABP4 protein. Following this functional blockade, we subsequently evaluated the protein expression of P21 and the senescence status of HTMCs. The experimental groups are as follows: (Jayaram et al., 2023): control group, (Benjafield et al., 2019), anti-FABP4 group, (Weinreb et al., 2014), PFOS group (20 μM), and (Ma et al., 2024) PFOS + anti-FABP4 group (20 μM).

### 2.15 Western blot

HTMC was lysed in pre-cooled RIPA lysis buffer (the ratio of RIPA, PMSF and phosphatase inhibitors:100:1:1). The total protein content of the lysate was determined by BCA method. Then, 20 μg of the total protein lysate was subjected to 10% SDS-PAGE electrophoresis, transferred onto nitrocellulose membrane, and blocked with 5% skimmed milk at 4 °C for overnight incubation. The primary antibodies included: FABP4 (1:1000) and P21 (1:1000). After the primary antibody incubation, the membrane was incubated at room temperature with IgG-HRP secondary antibody (1:5000) for 2 h. The protein bands were developed with ECL luminescence reagent and the gray value was measured using ImageJ software to quantify the protein expression level.

### 2.16 Statistical analysis

All statistical analyses were performed using R software (version 4.2.3). Continuous variables were compared between groups using Student's t-tests for normally distributed data or Wilcoxon rank-sum tests for non-normally distributed data. Categorical variables were analyzed using Pearson’s chi-square tests or Fisher’s exact tests, as appropriate. P value < 0.05 was considered statistically significant.

## 3 Results

### 3.1 Description of demographics and characteristic information of the study sample

According to the NHANES study design, a total of 20,497 participants were initially enrolled in this study. After applying exclusion criteria: primarily due to missing ophthalmic examination data or incomplete covariates, 1,165 eligible subjects were included in the final analysis, while 19,332 were excluded. As presented in Table 1, demographic and clinical characteristics were compared between participants with and without glaucoma. The number of glaucoma patients in the study population was = 118. Among all evaluated variables, except for Age, Marriage, PFOS, and Total alcohol, the other variables do not showed statistically significant. Of particular interest, serum PFOS concentrations were significantly higher in glaucoma patients (24.61 ± 2.26 ng/mL, p = 0.008) compared to non-glaucoma controls (19.31 ± 0.60 ng/mL).

**TABLE 1:** Demographics and characteristic of the non-glaucoma persons and glaucoma patients.

Characteristic	Overall, N = 1,165 (100%)^a^	NO glaucoma, N = 1,047 (89.87%)^a^	Glaucoma, N = 118 (10.13%)^a^	P Value^b^
Age	56.85 (0.48)	56.23 (0.48)	64.00 (1.83)	**<0.001**
Sex	​	​	​	0.2
Female	586 (52%)	531 (53%)	55 (44%)	​
Male	579 (48%)	516 (47%)	63 (56%)	​
Race	​	​	​	0.2
Non-Hispanic white	582 (73%)	523 (73%)	59 (74%)	​
Non-Hispanic black	221 (9.5%)	191 (9.1%)	30 (14%)	​
Mexican American	180 (6.1%)	166 (6.2%)	14 (4.8%)	​
Other Hispanic	129 (3.8%)	118 (3.9%)	11 (3.0%)	​
Other	53 (7.2%)	49 (7.5%)	4 (3.9%)	​
Education	​	​	​	0.4
9–11th grade	186 (12%)	166 (12%)	20 (14%)	​
College graduate or above	239 (30%)	218 (30%)	21 (27%)	​
High school Grad/GED	278 (25%)	253 (25%)	25 (25%)	​
Less than 9th grade	190 (7.8%)	165 (7.4%)	25 (13%)	​
Some college or AA degree	272 (26%)	245 (26%)	27 (21%)	​
PIR	3.15 (0.07)	3.16 (0.07)	3.07 (0.19)	0.4
Marriage	​	​	​	**<0.001**
Divorced	160 (13%)	147 (13%)	13 (10%)	​
Living with partner	39 (3.0%)	36 (3.2%)	3 (1.2%)	​
Married	678 (65%)	621 (66%)	57 (52%)	​
Never married	88 (6.7%)	80 (6.9%)	8 (5.0%)	​
Separated	45 (3.0%)	38 (2.5%)	7 (8.2%)	​
Widowed	155 (9.6%)	125 (8.4%)	30 (23%)	​
PFOS	19.74 (0.59)	19.31 (0.60)	24.61 (2.26)	**0.008**
BMI	28.98 (0.22)	29.01 (0.24)	28.66 (0.54)	0.9
Smoking.Status	​	​	​	0.11
Current	238 (20%)	222 (21%)	16 (13%)	​
Former	358 (28%)	308 (27%)	50 (37%)	​
Never	569 (52%)	517 (52%)	52 (50%)	​
Total alcohol	7.03 (0.74)	7.36 (0.80)	3.29 (1.32)	**0.021**

^a^ Mean.std.error (IQR) for continuous; n (%) for categorical.

^b^ Independent samples t-tests for continuous variables and chi-square tests for categorical variables.

The bold values indicate p < 0.05, which means they are statistically significant.

### 3.2 Multivariable analysis

As presented in Table 2, a multiple linear regression analysis was conducted to evaluate the association between serum PFOS concentrations and glaucoma. Model 2 demonstrated a significant positive association, with higher PFOS level correlating with glaucoma outcomes (Q2: β = 1.005 ± 0.210, p = 0.029; Q3: β = 1.099 ± 0.343, p = 0.026; Q4: β = 1.125 ± 0.391, p = 0.004). This dose-dependent relationship remained statistically significant after adjusting for all variables (Q2: β = 1.037 ± 0.423, p = 0.014; Q3: β = 1.111 ± 0.435, p = 0.010; Q4: β = 1.259 ± 0.399, p = 0.002). We also performed trend analysis and found the same trend that the adjusted odds of glaucoma were increased with greater serum PFOS concentrations in both models (model 2: p trend < = 0.008, model 3: p trend = 0.017).

**TABLE 2:** Multiple linear regression analysis on the association between PFOS and glaucoma.

PFOS	Model 1 OR (95% CI), p^a^	Model 2 OR (95% CI), p^b^	Model 3 OR (95% CI), p^c^
Q1	1.000	1.000	1.000
Q2	1.103 (0.298), p = 0.020	1.005 (0.210), p = 0.029	1.037 (0.423), p = 0.014
Q3	1.177 (0.311), p = 0.016	1.099 (0.343), p = 0.026	1.111 (0.435), p = 0.010
Q4	1.201 (0.369), p = 0.001	1.125 (0.391), p = 0.004	1.259 (0.399), p = 0.002
P for trend	0.007	0.008	0.017

^a^ Model 1: Unadjusted model.

^b^ Model 2: Adjusted for age, sex.

^c^ Model 3: Adjusted for age, sex, race, marriage, Total.alco, education, Smoking. Status, BMI, PIR.

OR, odds ratio; CI, confidence interval.

### 3.3 Epidemiology of glaucoma

**FIGURE 2:**
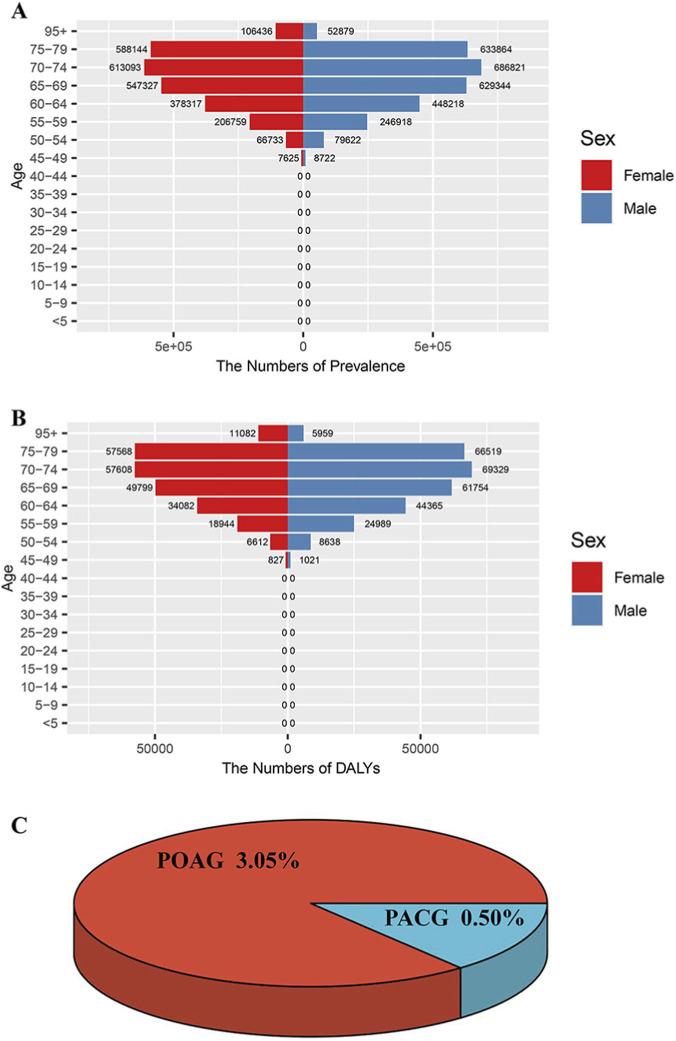
Epidemiology research of glaucoma **(A)** Age and gender characteristics of the global burden of glaucoma of prevalence in 2021 GBD database **(B)** Age and gender characteristics of the global burden of glaucoma of DALYs in 2021 GBD database. Red: female. Blue: male. Rows: number; columns: age **(C)** The prevalence of POAG and PACG. DALYs, disability-adjusted life years. POAG, primary open-angle glaucoma. PACG, primary angle-closure glaucoma.

In GBD database, glaucoma is more prevalent in peopleover the age of 40, and it increases quickly between the ages of 55 and 80. Males and females are most affected between the ages of 70 and 74. In terms of DALYs, similar tendencies are observed, with a substantial increase in incidence occurring after the age of 50. The highest of male DALYs peaks occurred between the ages of 70 and 74, while the highest of female DALYs peaks occurred between the ages of 70 and 79 (Figures 2A,B). The NHANES database stipulates that the age requirement for screening glaucoma patients should be above 40 years old. Our study also well explains that selecting patients in this age range is based on scientific grounds, demonstrating the rigor of our research. Due to limitations in accessing complete data from the GEO database, we conducted a literature review which revealed that POAG prevalence (3.05%) substantially exceeds that of PACG (0.50%) (Figure 2C). Consequently, we selected POAG as the representative condition for subsequent analyses.

### 3.4 Identification of blood PFOS targets affecting POAG

**FIGURE 3:**
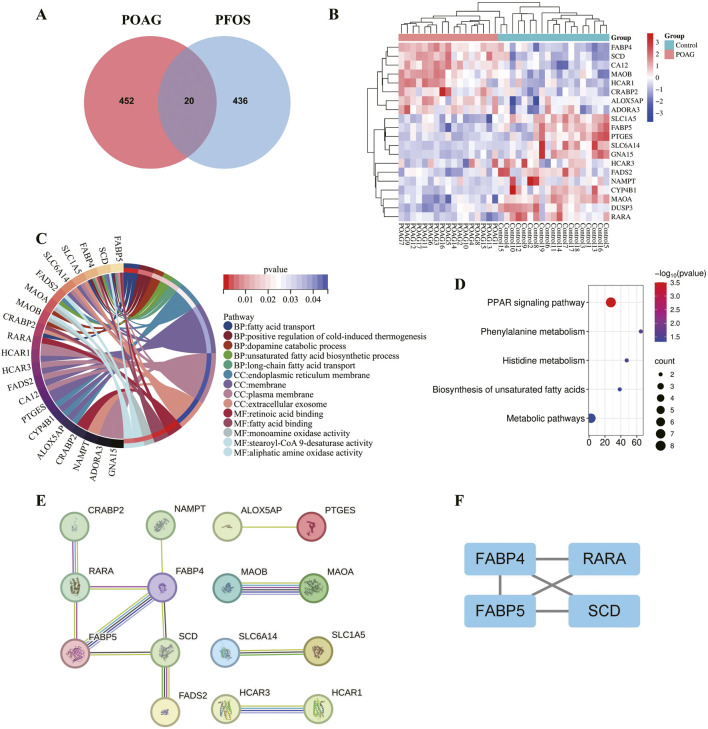
Identification of differentially expressed genes, functional enrichment analysis and PPI network analysis **(A)** Venn diagram of the genes of POAG and PFOS **(B)** The clustering heatmap of the expression pattern of 20 the most important differentially expressed genes. Rows: genes; columns: samples. Color scale represents normalized expression levels (red: upregulated; blue: downregulated), with intensity corresponding to fold-change magnitude **(C)** GO enrichment analysis **(D)** KEGG enrichment analysis. The larger the bubble, the more genes it represents. The redder the color, the smaller the p value **(E)** PPI network analysis by STRING database **(F)** PPI network analysis by MCC algorithm of Cytoscape. The number of lines between the two proteins represents the strength of the interaction.

There are significant differences in gene expression between POAG patients and healthy individuals. Compared with the control group, we extracted 472 differentially expressed genes from the GSE27276 dataset using R software, among which 259 were downregulated genes and 213 were upregulated genes (Supplementary Table S1). Meanwhile, by integrating the ChEMBL, TargetNet and SuperPred databases, we obtained a total of 456 PFOS-related non-redundant genes (Supplementary Table S2). The intersection of the genes regulated by POAG and PFOS is considered to be the key genes for PFOS-associated POAG, totaling 20 (Figure 3A). The clustering heatmap visually depicted the expression of 20 the most important differentially expressed genes between POAG group and control group. Among them, compared with the control group, the expression of FABP4 in the POAG group was significantly increased (Figure 3B).

### 3.5 Functional enrichment analysis

To further investigate the biological functions of the key genes, we conducted GO and KEGG enrichment analyses. GO analysis revealed that these genes are significantly associated with: BP: fatty acid transport, positive regulation of cold-induced thermogenesis, dopamine catabolic process, unsaturated fatty acid biosynthetic process and long-chain fatty acid transport. CC: endoplasmic reticulum membrane, membrane, plasma membrane and extracellular exosome. MF: retinoic acid binding, fatty acid binding, monoamine oxidase activity, stearoyl-CoA 9-desaturase activity and aliphatic amine oxidase activity (Figure 3C). KEGG pathway analysis indicated enrichment in several metabolic pathways, including the PPAR signaling pathway, biosynthesis of unsaturated fatty acids, and so on (Figure 3D). In summary, the GO and KEGG analyses primarily highlighted enrichment in lipid metabolism and energy metabolism. These findings provide valuable insights into the potential functional roles of these genes in metabolic regulation and disease mechanisms, warranting further investigation.

### 3.6 PPI network analysis

Using the STRING database and the MCC algorithm, a PPI network analysis was conducted on the 20 intersecting genes identified in the previous analysis. After excluding the genes with low interaction degrees, a comprehensive PPI network consisting of 4 nodes and 5 edges was constructed (Figures 3E,F).

### 3.7 Further screening of key genes through machine learning

**FIGURE 4:**
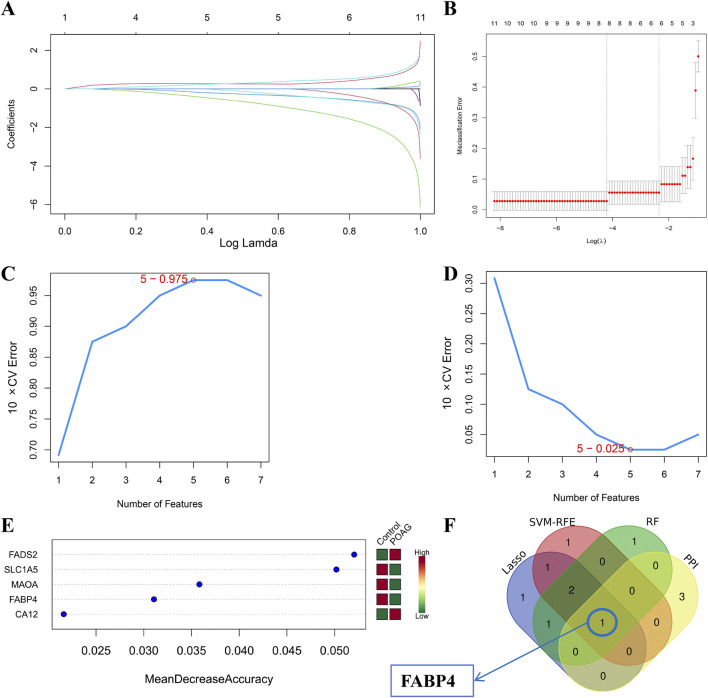
Machine learning **(A,B)** Lasso machine learning. In **(A)**, the different colored lines represent the 8 genes that were initially selected by Lasso. **(B)** further identified six key core genes based on the condition lambda. min = 6 **(C)** 10 × CV accuracy of SVM- RFE machine learning (maximal accuracy = 0.975) **(D)** 10×CV error of SVM-RFE machine learning (minimal RMSE = 0.025). The closer “maximal accuracy” is to 1 and “minimal RMSE” is to 0, the more reliable the result is. Generally, a maximal accuracy value greater than 0.7 indicates reliable results **(E)** Random Forest machine learning (top 5). The larger the MeanDecreaseAccuracy value is, the more important the gene is **(F)** Venn diagram of PPI network, Lasso, SVM- RFE and RF machine learning.

Next, we applied LASSO logistic regression with 10-fold cross-validation for feature selection, identifying 6 candidate core genes: FADS2, ALOX5AP, MAOA, DUSP3, FABP4 and SLC1A5 (Figures 4A,B). Penalty parameters were tuned by 10-fold cross validation, and 5 key genes using SVM-RFE algorithm: DUSP3, FABP4, PTGES, SLC1A5 and MAOA (Figures 4C,D). Random forest analysis showed that the genes with the five highest MeanDecreaseAccuracy scores included FADS2, SLC1A5, MAOA, FABP4 and CA12 (Figure 4E). A Venn diagram integrating the results from all methods ultimately identified one definitive core targets associated with PFOS-associated POAG: FABP4 (Figure 4F).

### 3.8 Molecular docking of PFOS-FABP4 interaction

**FIGURE 5:**
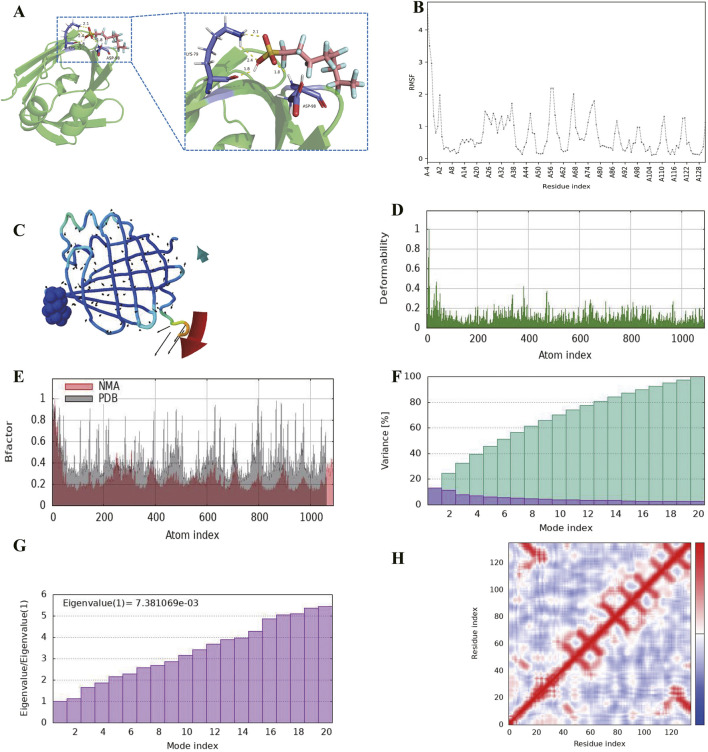
Molecular docking and molecular dynamics simulations of PFOS-FABP4 interaction **(A)** Molecular docking (the binding energies of PFOS-FABP4: −7.72 kcal/mol). Blue color represents the residues on the FABP4 protein, and pink color represents PFOS. The dotted lines indicate hydrogen bonds, and the longer the dotted lines are, the stronger the interaction is **(B)** RMSF plot of PFOS-FABP4 (average RMSF: 0.739 Å). The horizontal axis represents the residues on the FABP4 protein, while the vertical axis represents the stability of the binding between PFOS and FABP4. The smaller the fluctuations (the smaller the average value of RMSF) indicates a more stable binding **(C)** Molecular mobility analysis PFOS-FABP4. The small black arrows in the figure indicate the movement direction of individual amino acids, while the large arrows represent the overall movement trend of the protein (the longer arrows indicate larger displacements) **(D)** Deformability plot showing regions with potential structural flexibility. The peak value is moderate, indicating that it has a certain degree of flexibility and will not undergo excessive deformation, suggesting a good bond **(E)** B-factor comparison between theoretical (normal mode analysis, NMA) and experimental (PDB) fluctuations. There is little difference between NMA and PDB, indicating that the docking effect of PFOS and FABP4 is good **(F)** Variance plot showing the cumulative contribution of each normal mode to overall molecular motion. Colored bars show the individual (purple) and cummulative (green) variances **(G)** Eigenvalue plot Eigenvalue plot demonstrating motion stiffness. The lower values representing easier deformability **(H)** Covariance matrix of residue-pair motions plot. Correlated (red), uncorrelated (white), and anti-correlated (blue). There are more red areas, suggesting that the key amino acids in the protein move in a coordinated manner and the ligand binding is stable.

Based on the results of the above screening, PFOS was identified can target FABP4. Molecular docking analysis revealed a strong binding affinity between PFOS and FABP4, with a calculated binding energy of −7.72 kcal/mol, suggesting favorable intermolecular interactions. The stability of the PFOS-FABP4 complex was further supported by the formation of four hydrogen bonds between PFOS and key residues in the FABP4 binding pocket, specifically involving Lys79 and Asp98 (Figure 5A). Hydrogen bonding plays a critical role in mediating ligand-protein recognition, and these interactions likely contribute to the observed binding stability. These findings suggest that PFOS may competitively bind to FABP4, potentially disrupting its native biological function through direct molecular interaction.

### 3.9 Molecular dynamics simulations of PFOS-FABP4 interaction

CabsFlex dynamic simulation is used to verify and evaluate the stability of the ligand-receptor complex (PFOS-FABP4). After submitting the ligand-complex of the docking protein, CABS-Flex generates root mean square fluctuation (RMSF), and provides RMSF data and ten representative structural models. Figure 5 shows the resulting RMSF profile. The maximum RMSF of PFOS bound to FABP4 at residue 4 is 4.639 Å, the minimum RMSF at residue 105 is 0.107 Å, and the average RMSF is 0.739 Å, indicating relatively low structural fluctuations (Figure 5B). Fine molecular dynamics simulations were conducted using the iMODS server to evaluate the conformational mobility and structural stability of the ligand-protein complex. Normal mode analysis (NMA) was employed to study the collective motion and dynamic behavior of the ligand-protein complex (PFOS-FABP4). The 3D simulation dynamic result diagram shows the dynamic process of molecular docking (Figure 5C). In the deformable plot, it can be seen that the peak of the bonding area is moderate, indicating that it has a certain degree of flexibility but will not be overly distorted. This is a relatively good representation of the assembly result (Figure 5D). After docking, NMA and PDB fluctuated within a reasonable range, and there was not much difference between them. This indicates that the PFOS and FABP4 have a good docking effect and do not interfere with the natural movement pattern of the protein (Figure 5E). Next, the variance plot shows that the individual variance level is relatively high (about 12%), which indicates a low eigenvalue (Figure 5F). The eigenvalue represents the stiffness of the protein residue’s movement and shows the energy required for structural deformation. The eigenvalue obtained for this complex is 7.38 × 10^−3^, which can be regarded as low. This suggests that the interaction between PFOS and FABP4 is very good (Figure 5G). In the Covariance Map, there are more red areas, suggesting that the key amino acids in the protein move in a coordinated manner and the ligand binding is stable (Figure 5H). To validate ligand-induced stabilization, we compared the dynamic trajectories of the PFOS-FABP4 complex against the unliganded apo-FABP4 baseline. Comparative profiling revealed that PFOS binding markedly reduced the RMSF of key pocket residues, specifically Lys79 and Asp98. Furthermore, normal mode analysis demonstrated a higher eigenvalue for the complex (7.38 × 10^−3^) relative to apo-FABP4 (6.23 × 10^−3^), indicating enhanced overall structural stiffness and thermodynamic stability. Consistently, comparative deformability plots showed decreased flexibility in ligand-bound regions, corroborating the robust locking mechanism of PFOS.

### 3.10 Cell viability analysis of PFOS and H_2_O_2_

**FIGURE 6:**
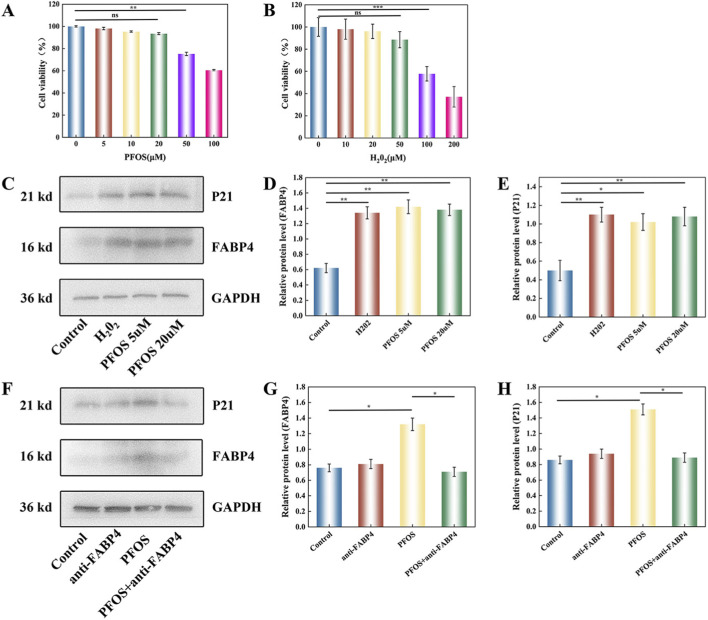
CCK8 assay and related protein expression **(A)** Cell viability of PFOS **(B)** Cell viability of H2O2 **(C–E)** The results of Western blot of FABP4 and P21 (n = 3) **(F–H)** The results of Western blot of FABP4 and P21 after targeted inhibition of FABP4 (n = 3). Note: *P < 0.05, **P < 0.01 and ***P < 0.001; ns, no significance; Results are shown as mean ± SD.

HTM cells were treated with different concentrations of PFOS and H_2_O_2_ (0, 5, 10, 20, 50, 100 and 200 μM) for 72 h. The results of the CCK8 assay showed that PFOS and H_2_O_2_ significantly reduced cell viability in a dose-dependent manner (Figures 6A,B), especially in cells exposed to 50 μM PFOS (p = 0.008) and 100 μM H_2_O_2_ (p = 0.0006) compared with the blank group, at which point the cell survival rate was below 80%, and a large number of HTMC cells died. Based on this finding, it is recommended to use 20 μM PFOS and 50 μM hydrogen peroxide for 72 h as the experimental standard for subsequent experiments.

### 3.11 The influence of PFOS on the expression levels of FABP4 and P21 proteins in HTMC

In order to make the experiment more convincing, we conducted a literature review and combined the results of previous experiments to establish the H_2_O_2_ group as the positive control group (POAG). Compared with the control group, both the PFOS 5 μM group and the PFOS 20 μM group showed that PFOS could increase the expression levels of FABP4 and P21 in HTMC (Figures 6C–E). Our study, which targeted and inhibited FABP4 using inhibitors, better demonstrates that PFOS promotes the expression of FABP4, leading to POAG. In the PFOS group, the expressions of FABP4 and P21 were significantly increased. Notably, while BMS-309403 is primarily designed to inhibit FABP4 protein function, our Western blot analysis revealed that this functional blockade also effectively attenuated the PFOS-induced upregulation of FABP4 and P21 protein levels (Figures 6F–H).

### 3.12 Detection of senescent cells with SA-β-gal assay

**FIGURE 7:**
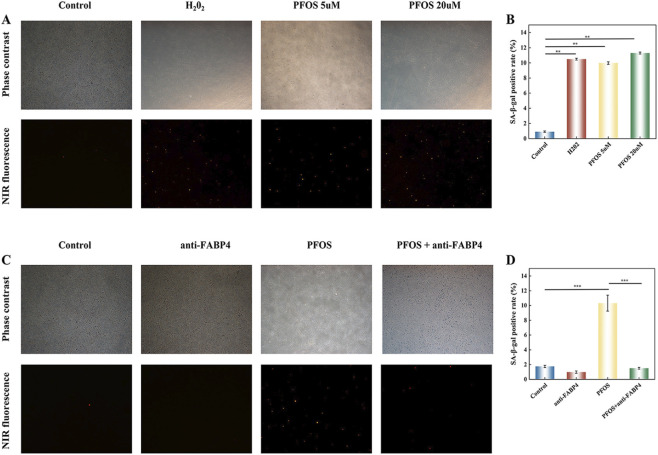
WDetection of senescent cells with SA-β-gal assay **(A)** Detection of senescent cells of PFOS **(B)** SA-β-gal positive rate of PFOS **(C)** Detection of senescent cells of PFOS after targeted inhibition of FABP4 **(D)** SA-β-gal positive rate of PFOS after targeted inhibition of FABP4. Note: **P < 0.01 and ***P < 0.001; Results are shown as mean ± SD.

Compared with the control group, the HTMC morphological models in the H_2_O_2_ group, the PFOS 5 μM group and the PFOS 20 μM group were polarized and deformed, and the number of senescent cells in HTMC significantly increased (Figures 7A,B). Targeted inhibition of FABP4 can better illustrate that PFOS can cause the aging of HTMC through FABP4. Compared with the blank group, the number of senescent cells in the PFOS group was significantly increased. However, after targeted inhibition of FABP4, this manifestation of aging could be reversed (Figures 7C,D).

## 4 Discussion

Our integrated investigation provides supporting evidence that PFOS exposure represents an environmental risk factor associated with glaucoma, which may contribute to POAG pathogenesis by promoting the premature senescence of human trabecular meshwork cells (HTMCs). By bridging epidemiological associations with *in vitro* mechanistic validation, we have elucidated a novel pathological axis: PFOS targets FABP4 to disrupt lipid homeostasis, which subsequently triggers the P21-mediated senescent phenotype in the trabecular meshwork. This cellular deterioration is a hallmark of POAG, as the accumulation of senescent HTMCs compromises the biophysical integrity of the outflow pathway, leading to impaired aqueous humor drainage.

Analysis of the NHANES cohort initially established a statistically significant, dose-dependent correlation between serum PFOS levels and glaucoma prevalence, a finding that remained robust after adjusting for traditional covariates. To our knowledge, this is the first study to identify PFOS as an independent environmental factor associated with Glaucoma. Furthermore, it is imperative to clarify the inherent constraints of the epidemiological dataset. The NHANES database relies on self-reported questionnaires for general ‘glaucoma’ status, rather than utilizing clinically confirmed specific diagnoses of POAG. Because POAG accounts for the vast majority of glaucoma cases globally, our subsequent transcriptomic and *in vitro* investigations were specifically designed to model POAG mechanisms. Crucially, given the cross-sectional nature of the NHANES survey design, our epidemiological analyses can only establish a significant statistical association between serum PFOS levels and glaucoma prevalence, rather than definitive causality. Consequently, we carefully posit that PFOS exposure is associated with an elevated risk of glaucoma and may contribute to POAG pathogenesis. The significance of this correlation is underscored by the biological role of the trabecular meshwork (TM) in maintaining intraocular pressure (IOP). In POAG, the TM undergoes progressive structural and functional decline, characterized by a transition toward cellular senescence. Our finding provides a necessary epidemiological foundation for investigating the direct toxicological impact of this “forever chemical” on TM cell longevity.

To decode the potential molecular triggers, we utilized machine learning and network toxicology approaches to pinpoint FABP4 as a pivotal candidate mediator. It is essential to note that these integrative bioinformatic findings are inherently hypothesis-generating. Nevertheless, this *in silico* analytical framework provided a robust, data-driven rationale for our subsequent *in vitro* investigations, which yielded vital empirical evidence supporting our computational predictions and suggesting a role for the PFOS-FABP4 axis in HTMC senescence. Molecular docking and dynamics simulations demonstrated that PFOS exhibits an exceptionally high binding affinity (ΔG = −7.72 kcal/mol) for the FABP4 hydrophobic pocket, inducing substantial conformational perturbations. As a specialized lipid chaperone, FABP4 is indispensable for the intracellular trafficking of fatty acid precursors required for the biosynthesis of unsaturated fatty acids (Furuhashi and Hotamisligil, 2008). Unsaturated fatty acids, such as docosahexaenoic acid (DHA), are not merely structural components but are vital for maintaining the membrane fluidity and mechanical elasticity required for HTMCs to respond to IOP fluctuations (Jacobs et al., 2021; Yarishkin et al., 2021). We propose that PFOS acts as a metabolic decoupler; by sequestering FABP4, it competitively inhibits the transport of endogenous lipids, thereby depriving the cell of the essential building blocks needed for membrane repair and homeostasis. This proposed mechanism strongly resonates with emerging toxicological breakthroughs, which demonstrate that environmental toxicants profoundly disrupt metabolic homeostasis and oxidative stress-responsive networks, often serving as critical upstream mediators for severe cellular dysfunction and disease progression in various tissues (Liu et al., 2023). By extending this broader paradigm of toxicant-induced metabolic disruption to the ocular microenvironment, our findings contextualize the PFOS-FABP4 axis not merely as an isolated ocular event, but as a specific, highly relevant manifestation of environmental metabolic toxicity, ultimately driving TM cell senescence and POAG progression.

The most critical evidence for this mechanism lies in our *in vitro* validation. Exposure to PFOS resulted in a significant increase in SA-β-gal activity—a gold-standard marker for senescence—alongside the upregulation of FABP4 and the cyclin-dependent kinase inhibitor P21 (Lee et al., 2006). The elevation of P21 is particularly decisive, as its levels at the end of the cell cycle determine the transition from transient arrest to permanent senescence and the initiation of a senescence-associated secretory phenotype (SASP) (Li et al., 2025). Crucially, the targeted inhibition of FABP4 was sufficient to reverse these effects, rescuing HTMCs from PFOS-induced senescence. This suggests that the “aging” of the trabecular meshwork under PFOS exposure is a directed toxicological process rather than a stochastic age-related decline. The accumulation of these non-functional cells likely leads to increased TM stiffness and a reduction in the effective filtration area, directly contributing to POAG pathogenesis. Beyond its specific role in mediating HTMC senescence elucidated in our study, FABP4 is canonically recognized as a critical regulator of broader metabolic and inflammatory pathways. Highly expressed in adipocytes and macrophages, elevated FABP4 is profoundly implicated in metabolic syndrome, insulin resistance, and atherosclerosis through the orchestration of pro-inflammatory responses (Furuhashi, 2019). In the context of our findings, this broader metabolic footprint suggests that PFOS-induced FABP4 overexpression may not only disrupt intracellular lipid homeostasis but could also foster a localized pro-inflammatory microenvironment within the trabecular meshwork, thereby synergistically compounding the pathogenesis of POAG (Li et al., 2021).

Several limitations of the present study warrant acknowledgment. First, the small clinical cohorts in both NHANES (n = 118) and the GSE27276 dataset (17 POAG, 19 controls) limit statistical power, rendering our bioinformatic predictions inherently hypothesis-generating. Second, regarding our *in vitro* mechanistic validation, a discrepancy exists between physiological serum levels and the supraphysiological PFOS dosages (5–20 μM) strategically employed as an accelerated model to elicit senescence within a 72-h timeframe. Furthermore, standard static cell cultures inherently fail to mimic the dynamic biomechanical environment of the physiological trabecular meshwork. Finally, the proposed PFOS-FABP4-P21 axis currently lacks validation in vivo animal models. Consequently, future investigations incorporating chronic low-dose exposures, biomechanically active 3D culture systems, and *in vivo* validation are essential to fully recapitulate lifelong environmental dynamics and structural complexities.

These mechanistic insights suggest promising therapeutic strategies. The use of FABP4 inhibitors, such as BMS309403, could prevent PFOS from interfering with endogenous lipid transport. Furthermore, our findings imply that exogenous supplementation of unsaturated fatty acids might bypass the transport blockade to maintain membrane integrity and delay HTMC senescence (Zhang et al., 2021; Kalogerou et al., 2022). While the cross-sectional nature of the NHANES data necessitates further longitudinal research, this study provides a novel theoretical framework: PFOS contributes to POAG development by modulating the FABP4-lipid axis to promote HTMC senescence.

## 5 Conclusion

This study identifies PFOS as a significant environmental risk factor for POAG, potentially contributing to disease progression through HTMC senescence. We demonstrate that PFOS targets FABP4 to disrupt unsaturated fatty acid biosynthesis, triggering cellular aging as evidenced by increased P21 and SA-β-gal activity. Crucially, inhibiting FABP4 reverses this PFOS-induced senescence, identifying the PFOS-FABP4 axis as a key mediator of trabecular meshwork dysfunction. These findings establish a novel mechanistic link between environmental pollutants and glaucoma, suggesting that targeting HTMC senescence is a promising strategy for POAG intervention.

## Data Availability

The original contributions presented in the study are included in the article/Supplementary Material, further inquiries can be directed to the corresponding authors.
