# Supplementary material for: Perfluorooctanesulfonic acid contributes to primary open-angle glaucoma in a FABP4-Dependent manner: a novel mechanism for environmental risk of glaucoma
Source: Front Genet. 2026 May 12;17:1807152. doi: 10.3389/fgene.2026.1807152 (PMC13200824; doi:10.3389/fgene.2026.1807152)
Supplement: Supplementary file 1 [file Table1.docx]

|  | **Table 1**. Demographics and characteristic of the non-glaucoma persons and glaucoma patients. | | | | |  |
| --- | --- | --- | --- | --- | --- | --- |
|  | **Characteristic** | Overall, N = 1165 (100%)1 | NO glaucoma, N = 1047 (89.87%)1 | Glaucoma,, N = 118 (10.13%)1 | P Value2 |  |
|  | Age | 56.85 (0.48) | 56.23 (0.48) | 64.00 (1.83) | **<0.001** |  |
|  | Sex |  |  |  | 0.2 |  |
|  | Female | 586 (52%) | 531 (53%) | 55 (44%) |  |  |
|  | Male | 579 (48%) | 516 (47%) | 63 (56%) |  |  |
|  | Race |  |  |  | 0.2 |  |
|  | Non-Hispanic White | 582 (73%) | 523 (73%) | 59 (74%) |  |  |
|  | Non-Hispanic Black | 221 (9.5%) | 191 (9.1%) | 30 (14%) |  |  |
|  | Mexican American | 180 (6.1%) | 166 (6.2%) | 14 (4.8%) |  |  |
|  | Other Hispanic | 129 (3.8%) | 118 (3.9%) | 11 (3.0%) |  |  |
|  | Other | 53 (7.2%) | 49 (7.5%) | 4 (3.9%) |  |  |
|  | Education |  |  |  | 0.4 |  |
|  | 9-11th Grade | 186 (12%) | 166 (12%) | 20 (14%) |  |  |
|  | College Graduate or above | 239 (30%) | 218 (30%) | 21 (27%) |  |  |
|  | High School Grad/GED | 278 (25%) | 253 (25%) | 25 (25%) |  |  |
|  | Less Than 9th Grade | 190 (7.8%) | 165 (7.4%) | 25 (13%) |  |  |
|  | Some College or AA degree | 272 (26%) | 245 (26%) | 27 (21%) |  |  |
|  | PIR | 3.15 (0.07) | 3.16 (0.07) | 3.07 (0.19) | 0.4 |  |
|  | Marriage |  |  |  | **<0.001** |  |
|  | Divorced | 160 (13%) | 147 (13%) | 13 (10%) |  |  |
|  | Living with partner | 39 (3.0%) | 36 (3.2%) | 3 (1.2%) |  |  |
|  | Married | 678 (65%) | 621 (66%) | 57 (52%) |  |  |
|  | Never married | 88 (6.7%) | 80 (6.9%) | 8 (5.0%) |  |  |
|  | Separated | 45 (3.0%) | 38 (2.5%) | 7 (8.2%) |  |  |
|  | Widowed | 155 (9.6%) | 125 (8.4%) | 30 (23%) |  |  |
|  | PFOS | 19.74 (0.59) | 19.31 (0.60) | 24.61 (2.26) | **0.008** |  |
|  | BMI | 28.98 (0.22) | 29.01 (0.24) | 28.66 (0.54) | 0.9 |  |
|  | Smoking.Status |  |  |  | 0.11 |  |
|  | Current | 238 (20%) | 222 (21%) | 16 (13%) |  |  |
|  | Former | 358 (28%) | 308 (27%) | 50 (37%) |  |  |
|  | Never | 569 (52%) | 517 (52%) | 52 (50%) |  |  |
|  | Total alcohol | 7.03 (0.74) | 7.36 (0.80) | 3.29 (1.32) | **0.021** |  |
|  | 1 Mean.std.error (IQR) for continuous; n (%) for categorical | | | | |  |
|  | 2 Independent samples t-tests for continuous variables and chi-square tests for categorical variables. | | | | |  |
