# Supplementary figures and images for: Perfluorooctanesulfonic acid contributes to primary open-angle glaucoma in a FABP4-Dependent manner: a novel mechanism for environmental risk of glaucoma
Source: Front Genet. 2026 May 12;17:1807152. doi: 10.3389/fgene.2026.1807152 (PMC13200824; doi:10.3389/fgene.2026.1807152)

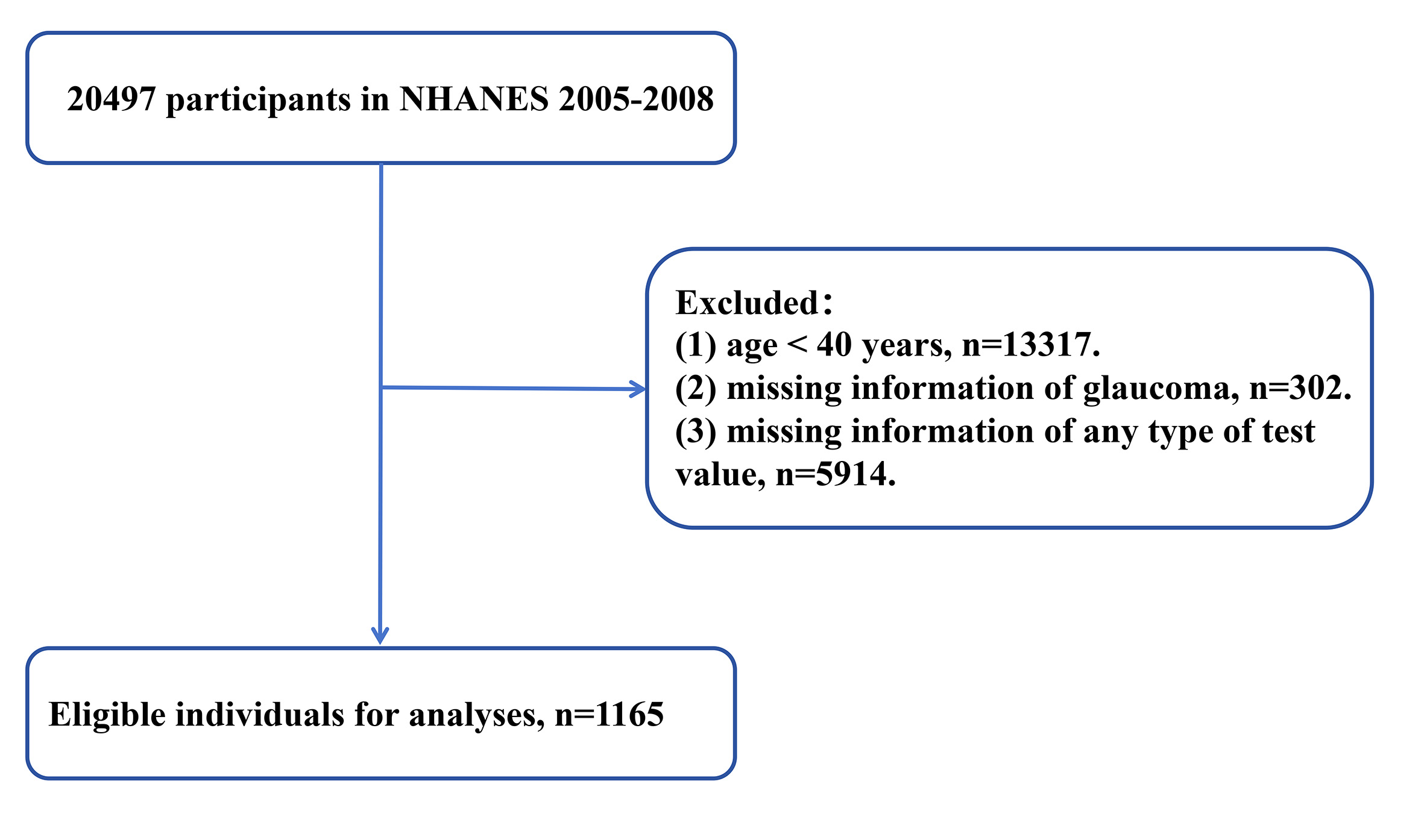

Supplement: Supplementary file 2 [file Image1.jpeg]
