# Supplementary material for: Perfluorooctanesulfonic acid contributes to primary open-angle glaucoma in a FABP4-Dependent manner: a novel mechanism for environmental risk of glaucoma
Source: Front Genet. 2026 May 12;17:1807152. doi: 10.3389/fgene.2026.1807152 (PMC13200824; doi:10.3389/fgene.2026.1807152)
Supplement: Supplementary file 3 [file Table2.docx]

|  | **Table 2.** Multiple linear regression analysis on the association between PFOS and glaucoma. | | | |  |
| --- | --- | --- | --- | --- | --- |
|  | **PFOS** | **Model 1 OR (95% CI), p**1 | **Model 2 OR (95% CI), p**2 | **Model 3 OR (95% CI), p**3 |  |
|  | Q1 | 1.000 | 1.000 | 1.000 |  |
|  | Q2 | 1.103（0.298）, p=0.020 | 1.005（0.210）, p=0.029 | 1.037（0.423）, p=0.014 |  |
|  | Q3 | 1.177（0.311）, p=0.016 | 1.099（0.343）, p=0.026 | 1.111（0.435）, p=0.010 |  |
|  | Q4 | 1.201（0.369）, p=0.001 | 1.125（0.391）, p=0.004 | 1.259（0.399）, p=0.002 |  |
|  | P for trend | 0.007 | 0.008 | 0.017 |  |
|  | 1 Model 1: unadjusted model. | | | |  |
|  | 2 Model 2: adjusted for age, sex. | | | |  |
|  | 3 Model 3: adjusted for age, sex, race, marriage, Total.alco, education, Smoking.Status, BMI, PIR.  OR, odds ratio; CI, confidence interval. | | | |  |
